# Depressive symptoms among women in Raqqa Governorate, Syria: associations with intimate partner violence, food insecurity, and perceived needs

**DOI:** 10.1017/gmh.2019.20

**Published:** 2019-10-02

**Authors:** K. L. Falb, A. Blackwell, J. Stennes, M. Hussein, J. Annan

**Affiliations:** 1 Research, Evaluation & Learning, International Rescue Committee, 1730 M St NW, Ste 505, Washington DC 20036, USA; 2 Women's Protection & Empowerment, International Rescue Committee, 3 Bloomsbury Place, London WC1A 2QL, UK; 3 Women's Protection & Empowerment, International Rescue Committee, Dohuk, Iraq; 4 Research, Evaluation & Learning, International Rescue Committee, 122 E 42nd St, NYC, NY 10068, USA

**Keywords:** Depression, food insecurity, gender-based violence, humanitarian, Syria

## Abstract

**Background.:**

Raqqa Governorate, Syria has recently been affected by overlapping conflicts related to the Syrian Civil war and occupation by ISIS, resulting in widespread displacement and disruption of economic livelihoods. However, little information is currently known about mental health needs and risk factors among women. Therefore, this study sought to examine potential risk factors for depressive symptoms among married women living in northern Syria.

**Methods.:**

Data were collected between March and April 2018 as part of an evaluation of an International Rescue Committee cash transfer program targeted toward vulnerable households. Using cross-sectional data from 214 married women participating in the program, linear regression models were generated to explore the associations between depressive symptoms [nine-item Patient Health Questionnaire (PHQ-9)] and its potential risk factors, including food insecurity, perceived deprivation of basic needs [the Humanitarian Emergency Settings Perceived Needs Scale (HESPER) scale], and past-3-month intimate partner violence (IPV).

**Results.:**

The average depressive symptom score was 10.5 (s.d.: 4.9; range: 2–27). In the final adjusted model, any form of recent IPV (*β* = 2.25; 95% CI 0.92–3.57; *p* = 0.001), severe food insecurity (*β* = 1.62; 95% CI 0.27–2.96; *p* = 0.02) and perceived needs (*β* = 0.38; 95% CI 0.18–0.57; *p* = 0.0002) were associated with an increase in depressive symptoms.

**Conclusion.:**

Study findings point to the need to address the mental health needs of women in conflict-affected areas of Syria. Programming to address risk factors for depression, including IPV and other factors associated with daily stressors such as food insecurity and deprivation of basic needs, may be effective in reducing depression in this population.

## Introduction

Over the last 7 years, dual crises from civil conflict and ISIS occupation have left the population of northern Syria exposed to extreme deprivation and violence. Despite the cessation of ISIS occupation and an ebbing of civil conflict in 2018, communities within the northern region of Raqqa Governorate continue to suffer. While over 150 000 individuals have returned to Raqqa City, living conditions are substandard due to high levels of destruction, and many families continue to live in internally displaced person (IDP) sites or in other informal settlements with little access to economic livelihoods (UN Office for the Coordination of Humanitarian Affairs, 2018).

In addition to the direct experiences of war, the indirect, economic-related experiences of conflict have serious implications for the health and wellbeing of affected populations. Deprivation, loss of income and assets and subsequent poverty are significant risk factors for poor mental health, including both post-traumatic stress disorder (PTSD) and substance abuse disorders (Ezard, 2012; Kazour *et al*. 2017). Dispossession, including losing one's property, job or livelihood, creates economic and psychological stress for households that can negatively impact the mental health of the entire family. Poverty is also a risk factor for mental health issues as an underlying factor of malnutrition, stress, substance abuse, social exclusion and exposure to trauma and violence (Lund *et al*. 2010; Sacks *et al*. 2010). With the destruction of infrastructure and the disruption of social norms and institutions, these economic stressors are exacerbated among conflict-affected populations. Over time, lack of education, lack of assets, inability to work and insecure living conditions have devastating impacts on the mental health of conflict-affected individuals and weaken the resilience of their households and communities far beyond the end of the conflict (Roberts & Browne, 2011).

Indeed, a recent literature review of mental health issues amongst Syrians affected by armed conflict suggest that increased risk of poor mental health may be the result of challenges adapting to the post-emergency context and problems related to conflict and displacement (Hassan *et al*. 2016), as the daily psychological stress experienced by conflict-affected populations can exacerbate poor mental health (Miller & Rasmussen, 2010; Perkins *et al*. 2018). Increased risk of poor mental health may arise due to prolonged exposure to psychological stress from traumatic experiences including deprivation of shelter, food and other basic needs, as well as forced displacement, witnessing destruction of residential areas, and direct experiences of violence, torture, exploitation and abuse (Roberts & Browne, 2011; Lindgren *et al*. 2012; Lindert *et al*. 2016).

Due to the combined impact of these direct and indirect conflict-related stressors, conflict-affected populations have an elevated prevalence of major depressive disorder and PTSD; a meta-analysis of studies in conflict-affected populations found that the pooled prevalence of major depression was more than two times higher and PTSD was more than three times higher in conflict-affected populations than the global mean prevalence of these disorders (Charlson *et al*. 2016). This is apparent among Syrian refugees, for whom persistent instability, displacement, and conflict have resulted in high levels of depressive symptoms (M'zah *et al*. 2019; Poole *et al*. 2018; Tekeli-Yesil *et al*. 2018). Recent studies conducted among refugees from the northern region of Syria have found high prevalence of depressive symptoms (from 37% to 44%) and post-traumatic stress symptoms (from 33.5% to 38%) among Syrian refugees (Alpak *et al*. 2015; Naja *et al*. 2016; Ibrahim & Hassan, 2017; Kazour *et al*. 2017; Acarturk *et al*. 2018). Consistent with global literature, the prevalence of mental health concerns among these populations is magnified among women (Hollander *et al*. 2011; Roberts & Browne, 2011; Charlson *et al*. 2016). For instance, amongst Syrian refugees in Greece, women were over three times more likely to have depressive symptoms than men (Poole *et al*. 2018). Additionally, amongst children still living in Syria, nearly one-third met criteria for probable depression, with girls at heightened risk (Perkins *et al*. 2018).

An underlying hypothesis behind these higher rates of depressive symptoms among women and girls in conflict settings is that mental health is often related to experiences of gender-based violence (GBV), including intimate partner violence (IPV) and non-partner sexual violence (Roberts & Browne, 2011; Stark & Ager, 2011; Charlson *et al*. 2016). Ongoing daily experiences with IPV may be particularly problematic as IPV has consistently been linked with poor physical and mental health outcomes in non-emergency settings (Campbell, 2002; Ellsberg *et al*. 2008). Further, recent research suggests that experiencing IPV confers additional risk for poor mental health among conflict-affected women, in addition to the mental health impact of trauma related to crises (Falb *et al*. 2013*a*; Gupta *et al*. 2014).

Few rigorous studies have been conducted among populations still within Syria, and those that have been conducted do not examine the specific needs and issues of women and girls. More evidence is therefore needed on these populations.

This paper aims to explore the depressive symptomatology amongst women currently residing in Raqqa Governorate, Syria. It was hypothesized that metrics related to daily stressors, including higher food insecurity and perceived household needs, and experiences of different types of IPV will be associated with higher depressive symptomatology. By identifying the associations between economic stressors, IPV and depression, we can increase our understanding of the experiences of women in acute emergencies to better develop and target mental health interventions and other programming that might affect mental health.

## Methods

### Survey design

Data are from a baseline survey collected in March and April of 2018 as part of an assessment of cash transfer programming by the IRC, which is a non-governmental humanitarian organization that provides protection, economic, health, and other services in conflict-affected areas in Northeast Syria. The target population for the cash transfer program was not based in IDPs camps, but rather in villages and towns throughout Northeast Syria. This setting included both those who were displaced by airstrikes and fighting from Raqqa City and also those who remained in their homes during the conflict. In addition, ISIS had recently withdrawn from the towns, thereby increasing access for humanitarian organizations to reach and deliver programming to affected populations living within the towns.

A total of 712 households in the selected programmatic villages were assessed by IRC to determine cash program eligibility. A total of 693 households (97.3% of the accessible population) were eligible to receive cash based on pre-defined socioeconomic vulnerability criteria, which included economic variables as well as social factors such as household composition. Among these, all households with at least one woman age 18–59 were invited to participate in the survey (596 eligible households). In female-headed households, the head of household was invited to participate. In male-headed households containing more than one eligible woman 18–9, a Kish grid was used to randomly select the respondent among eligible women. A total of 512 interviews were completed, for a response rate of 85.9%. Given the focus on IPV, the present analysis was restricted to partnered women (*N* = 214; 42.8% of the sample) as the full study sample for the cash transfer program evaluation included married, single, divorced, and widowed women.

### Data collection

Female enumerators were identified in the communities and contracted by the IRC monitoring and evaluation team specifically for this study. All data collectors received training on GBV research ethics and interview skills. Surveys were conducted in private spaces within interview sites by female enumerators who were not from the same community as the respondent. Verbal informed consent was completed prior to beginning the survey. Consent forms included specific language to clarify that the women were still eligible to participate in the cash transfer program regardless of whether or not they participated in the evaluation study. Study tools were translated into Arabic, piloted, and back-translated into English. Data were then collected using a CSPro data collection and entry platform on phones. Women were referred to IRC's Women's Protection and Empowerment case managers at the conclusion of the survey if the respondent wished to receive additional support. All survey procedures were approved by IRC's Institutional Review Board and access approval was received by local authorities, including the Humanitarian Access Office.

### Measures

The primary dependent variable, *depression score*, was calculated using the nine-item Patient Health Questionnaire (PHQ-9) (Kroenke *et al.* Spitzer, & Williams, 2001; AlHadi *et al*. 2017). Each item is given a value of 0–3 reflecting how often the respondent has experienced each depression symptom in the past 2 weeks: not at all (0), several days (1), more than half the days (2), or nearly every day (3). All items are totaled to create a single score ranging from 0 to 27. Cronbach's *α* of the PHQ-9 within this dataset was 0.72, indicating acceptable reliability. Based on the PHQ scale, scores ranging 5–9 indicate mild depressive symptoms, 10–14 indicate moderate depressive symptoms, and 15–19 and 20+ indicate moderately severe depression and severe depression, respectively. These cutoffs were not validated in this study but the scale has been used in populations of Syrian and other Arabic-speaking asylum seekers in displacement camps in Germany and Greece (Georgiadou *et al*. 2017; Poole *et al*. 2018). These cut-offs are used in this study to aid in the descriptive understanding of reported symptoms in comparison to other settings; however, depressive symptoms are modeled continuously and the study does not aim to provide a clinical diagnosis.

Household *perceived needs* were measured using an adapted version of the World Health Organization's Humanitarian Emergency Settings Perceived Needs Scale (HESPER) (WHO, 2011). This scale asks whether the respondent feels that she has a ‘serious problem’ regarding 20 different items that are relevant to basic needs in a humanitarian setting, including basic materials, physical health, safety, education, and social issues. The summary variable is a continuous measurement of the number of times a respondent perceived an issue to be a serious problem.

*Severe food insecurity* was measured using the Household Food Insecurity Access Scale (HFIAS) (Coates *et al*. 2007). This scale consists of nine questions on food quantity and quality for the respondent's household in the past 4 weeks. Households are coded as experiencing severe food insecurity if they reported experiencing any item on the HFIAS scale often (more than 10 days in the past 4 weeks). This was selected due to the high levels of food insecurity within the population.

Experience of IPV in the past 3 months was assessed using the WHO Multi-country Study on Domestic Violence & Women's Health modules (WHO, 2005). *Physical IPV* includes women who reported that their husband has slapped, hit, choked, pushed, shoved, kicked, dragged, choked, burned, or used a weapon against them in the past 3 months. *Sexual IPV* includes women who reported that their husband has forced them to have sex in the past 3 months. This includes coercion with threats and intimidation as well as physical force. *Emotional abuse* includes women who report that their husband has insulted, intimidated, threatened, belittled, or humiliated them in the past 3 months. If a woman affirmatively responded to any of the items, they were coded as experiencing that form of IPV. Forms of IPV were combined for the final predictor variable of any physical, sexual, or emotional IPV in the past 3 months.

Disability status was assessed using the Washington Group on Disability Statistics Short Set Questions (Washington Group on Disability Statistics, 2016). This set of six questions identifies those individuals with a greater risk than the general population for participation restrictions based on the presence of difficulties in doing six core functions. Participants were labeled as having a disability if they reported having some or a lot of difficulty doing the core functions or could not do them at all. These core functions include seeing, hearing, walking or climbing steps, remembering or concentrating, washing or dressing, and communicating in their usual language. The summary variable is a scale of the severity of any disability reported by an individual (mild or none, moderate or severe). Age in years was continuously assessed. Additional categorical variables included ever attended school, current livelihood status, and current displacement status. For livelihoods, categories included housewife/unemployed, daily laborer (signifying unstable employment in an unspecified occupation), and other (a collapsed variable of ‘famer, ‘trader’, ‘business owner’, and ‘other’).

### Data analysis

Descriptive statistics were generated and correlations were tested using χ^2^ or *t* tests. Missing items (0.6%) of scales were coded as 0 to retain statistical power and for conservative estimates. Unadjusted linear regressions were constructed for the predictor variables and demographics of interest, which included ever attended school, current livelihood, current displacement status, and disability. Adjusted linear regressions were constructed for each predictor variable (perceived needs, severe food insecurity, and IPV) in separate models, controlling for demographics listed above. The final model included all three predictor variables. All analyses were completed in SAS 9.4.

## Results

### Sample characteristics

Demographic characteristics are presented in Table 1. In the study sample, over half (55.6%) of women had ever attended school and one-third (33.6%) were currently displaced. Twenty-eight percent of women reported some form of disability and three-quarters (77.2%) were unemployed. The average age of women was 35.4 years (s.d.: 9.2).

**Table 1.** Frequencies or means of demographic characteristics, by perceived needs, severe food insecurity, past-3-month IPV status, and depressive symptom score (*N* = 214)

	Total^a^	Perceived needs Mean (s.d.)	Severe food insecurity % (*N*)	Any physical/sexual/emotional IPV % (*N*)	Physical IPV % (*N*)	Sexual IPV % (*N*)	Emotional IPV % (*N*)	Depression symptom score (s.d.)
Total	–	12.2 (3.4)	67.8% (145)	33.2% (71)	14.0% (30)	16.8% (36)	24.8% (53)	10.5 (4.9)
Age^b^								
18–25	16.3% (34)	12.4 (3.5)	58.8% (20)	52.9% (18)	26.5% (9)	29.4% (10)	38.2% (13)	12.0 (6.1)
26–35	36.8% (77)	12.2 (3.2)	63.6% (49)	28.6% (22)	14.3% (11)	14.3% (11)	18.2% (14)	10.3 (4.8)
36+	46.9% (98)	12.0 (3.5)	72.5% (71)	29.6% (29)	10.2% (10)	14.3% (14)	24.5% (24)	10.1 (4.4)
Education								
Ever attended school	55.6% (119)	12.3 (3.2)	67.8% (79)	39.5%* (47)	17.7% (21)	21.1% (25)	29.4% (35)	10.3 (4.9)
Never attended school	44.4% (95)	12.0 (3.5)	69.5% (66)	25.3% (24)	9.5% (9)	11.6% (11)	19.0% (18)	10.8 (4.9)
Current livelihood^c^								
Housewife/unemployed	77.2% (156)	12.1 (3.4)	62.8% (98)	30.8% (48)	14.1% (22)	13.5% (21)	23.1% (36)	9.8 (4.7)
Daily laborer	14.4% (29)	12.7 (3.5)	69.0% (20)	31.0% (9)	10.3% (29)	20.7% (6)	20.7% (6)	12.3 (5.3)
Other	8.4% (17)	13.2 (2.6)	88.2% (15)	47.1% (8)	17.7% (3)	23.5% (4)	35.3% (6)	11.2 (4.5)
Displacement status								
Currently displaced	33.6% (72)	13.3** (2.5)	63.9% (46)	40.3% (29)	15.3% (11)	20.8% (15)	33.3%* (24)	10.8 (5.0)
Not currently displaced	66.4% (142)	10.4 (4.8)	69.7% (99)	29.6% (42)	13.4% (19)	14.8% (21)	20.4% (29)	10.4 (4.8)
Disability status								
Disability	28.0% (60)	12.9* (3.4)	78.3%* (47)	35.0% (21)	8.3% (5)	20.0% (12)	26.7% (16)	12.4*** (5.4)
No disability	72.0% (154)	11.9 (3.3)	63.6% (98)	32.5% (50)	16.2% (25)	15.6% (24)	24.0% (37)	9.8 (4.4)

a Overall cells are column percentages; remaining cells are row percentages.

b Five missing data.

c Twelve missing data.

*p < 0.05; **p < 0.01; ***p < 0.0001.

The average depression symptom score was 10.5 (s.d.: 4.9; range: 2–27). Based on the PHQ scale, 40.2% of women reported mild depressive symptoms (PHQ9: 5–9), 26.6% reported moderate depressive symptoms (PHQ9: 10–14), 20.6% reported moderately severe depressive symptoms (PHQ9:15–19); and 3.7% reported severe depressive symptoms (PHQ: 20+).

Mean perceived needs was 12.2 (s.d.: 3.4; range 2–20) and was significantly associated with currently being displaced and reporting some form of disability. Nearly seven out of 10 (67.8%) women reported any form of severe food insecurity; this was significantly associated with reporting some form of disability. One-third (33.2%) reported any form of physical, sexual, and emotional IPV. The most common form of IPV was emotional IPV, followed by sexual IPV and physical IPV.

### Unadjusted associations with depressive symptoms

In unadjusted linear regression models presented in Table 2, one additional unit change in perceived needs was associated with an increase in depressive symptoms (*β* = 0.58; 95% CI 0.29–0.66; *p* < 0.001). Reporting severe food insecurity in the past month was also associated with an increase in depressive symptoms (*β* = 2.96; 95% CI 1.62–4.31; *p* < 0.001). Any past-3-month IPV (*β* = 2.81; 95% CI 1.47–4.15; *p* < 0.001); emotional IPV (*β* = 3.63; 95% CI 2.19–5.07; *p* < 0.001); and sexual IPV (*β* = 2.42; 95% CI 0.70–4.14; *p* < 0.001) were associated with increases in depressive symptoms; while past-3-month physical IPV did not show a significant association. Demographics associated with increased risk of depressive symptoms in unadjusted models included being a daily laborer and having a disability.

**Table 2.** Unadjusted and adjusted associations of perceived needs, severe food insecurity, IPV, and demographics with depression score (*N* = 197)

	Unadjusted models *β* (95% CI)	Model 1. Full adjusted model *β* (95% CI)	Model 2. Full adjusted model *β* (95% CI)
Perceived needs	0.47 (0.29–0.66)***	0.38 (0.18–0.57)**	0.31 (0.10–0.51)**
Severe food insecurity	2.96 (1.62–4.31)***	1.62 (0.27–2.96)*	1.75 (0.41–3.09)**
Past 3 months physical, sexual, and emotional IPV	2.81 (1.47–4.15)***	2.25 (0.92–3.57)***	
Past 3 months physical IPV	1.07 (−0.81 to 2.95)	–	−0.72 (−2.73 to 1.30)
Past 3 months sexual IPV	2.42 (0.70–4.14)**	–	0.29 (−1.54 to 2.12)
Past 3 months emotional IPV	3.63 (2.19–5.07)***	–	3.03 (1.27–4.80)**
*Demographic*			
Age	−0.07 (−0.14 to 0.007)	−0.07 (−0.15 to −0.002)*	−0.09 (−0.16 to −0.02)*
Ever attended school	−0.48 (−1.79 to 0.84)	−1.39 (−2.73 to −0.04)*	−1.40 (−2.73 to −0.06)*
Current livelihood			
Housewife/unemployed	Ref	Ref	Ref
Daily laborer	2.08 (0.43–4.22)*	1.66 (−0.06 to 3.39)	1.73 (0.00009–3.46)*
Other	0.84 (−1.36 to 3.49)	0.07 (−2.11 to 2.25)	0.12 (−2.05 to 2.29)
Currently displaced	0.38 (−1.01 to 1.76)	−0.24 (−1.59 to 1.12)	−0.19 (−1.54 to 1.16)
Disability	2.62 (1.20–4.04)**	2.22 (0.86–3.58)**	2.21 (0.85–3.59)**
*R*^2^	–	0.29	0.30

*p < 0.05; **p < 0.01; ***p < 0.001.

### Adjusted associations with depressive symptoms

Table 2 also presents two adjusted linear models. The first model includes the predictors of interest – perceived needs, severe food insecurity, and any form of past-3-month IPV, along with demographics including age, school attendance, livelihood, displacement status, and disability. In this model, any form of past-3-month IPV (*β* = 2.96; 95% CI 1.62–4.31; *p* < 0.0001), severe food insecurity (*β* = 1.62; 95% CI 0.27–2.96; *p* = 0.02), and perceived needs (*β* = 0.38; 95% CI 0.18–0.57; *p* = 0.0002) were associated with an increase in depressive symptoms.

The second adjusted model provides further nuance by including a breakdown of IPV into emotional, physical, or sexual IPV. Perceived needs and severe food insecurity, along with demographics listed above, are also included in this Model 2. In this adjusted model, past-3-month emotional IPV was associated with an increase in depressive symptoms (*β* =  3.03; 95% CI 1.27–4.80; *p* = 0.0009), while past-3-month physical and sexual IPV were not significantly associated with the outcome of interest. Severe food insecurity (*β* = 1.75; 95% CI 0.41–3.09; *p* = 0.01) and perceived needs (*β* = 0.31; 95% CI 0.10–0.51; *p* = 0.003) were still significantly associated with an increase in depressive symptoms. Older age (*β* = −0.09; 95% CI −0.16 to −0.02) and attending school (*β* = −1.40; 95% CI −2.73 to −0.06) were associated with reduced depressive symptoms in the final model. Being a daily laborer (*β* = 1.73; 95% CI 0.00009–3.46) and having a disability (*β* = 2.21; 95% CI 0.85–3.59) were significantly associated with increased depressive symptoms in the final model.

## Discussion

This study sought to examine depressive symptomatology amongst married women in Raqqa Governorate, Syria and its associations with IPV and metrics related to daily stressors, such as food insecurity and perceived household needs. Findings demonstrate that the average depressive symptom score of women residing in Raqqa Govenorate was 10.5, which is within the range of moderate depression (e.g. 50.9% of women met criteria for at least moderate depression) (Kroenke *et al*. 2001). The average score was substantially higher than other conflict settings such as in the Republic of Georgia (Comellas *et al*. 2015), but also slightly lower than what was found in other settings such as Darfur, Sudan (Kim *et al*. 2007), Somali refugees in Ethiopia (Feyera *et al*. 2015), or South Sudanese women in Uganda (Tol *et al*. 2018). Rigorous research regarding mental health concerns amongst this population is lacking; however, this study adds to the evidence pointing to the high levels of depressive symptoms that occur, both within Syria and in refugee populations, despite differing contextual factors including the setting in which individuals and families are residing (Georgiadou *et al*. 2017; Sijbrandij *et al*. 2017; Tinghög *et al*. 2017). For instance, a study comparing the determinants of mental disorders in Syrian refugees in Turkey to Syrian IDPs found that major depressive disorder was highly prevalent in both populations (58.8% among IDPs and 70.5% among refugees). The study also found that post-migration factors (including economic status, working status, receipt of economic support, and family unity after migration, among others) were stronger predictors of conflict-related mental health than pre-migration economic factors for both settings (Tekeli-Yesil *et al*. 2018).

In systematic reviews and meta-analyses, IPV has been consistently linked to increased risk of depression (Beydoun *et al*. 2012; Devries *et al*. 2013; Bacchus *et al*. 2018). Similarly, in the present study, we also document a robust relationship between depressive symptoms and the overall score for any form of past-3-month physical, sexual, and emotional IPV. This relationship was driven by emotional IPV as the most salient predictor of depressive symptoms in the final model, as physical and sexual IPV fell to non-significance.

The associations between different forms of IPV and depressive symptoms seem to vary across populations and contexts. For instance, in a South African population, only emotional and sexual IPV was associated with depression, after controlling for covariates (Okafor *et al*. 2018). Alternatively, in a population in Afghanistan, emotional IPV by itself was not associated with IPV (Gibbs *et al*. 2018*a*). In a recent study conducted in informal settlements in South Africa, women who experienced only emotional or economic IPV reported more depressive symptoms than those who experienced physical or sexual IPV only (Gibbs *et al*. 2018*b*). Importantly, the study also revealed that the highest levels of depression were seen among women who experienced emotional or economic IPV combined with physical or sexual IPV. Given emotional IPV's strong overlap with physical, sexual, and economic violence, it is difficult to assess independently and therefore evidence on its individual association remains limited. However, women experience emotional abuse in many different forms and often experience it pervasively and subconsciously compared with the more discrete acts of other forms of IPV (Jewkes, 2010), and therefore may be important to consider for screening and programming in low-resource or high-risk settings, such as in conflict-affected populations.

Daily stressors related to residing in a conflict-affected setting, operationalized as severe food insecurity and perceived needs, were also significantly correlated with increased risk of depressive symptoms across all models. Food insecurity, in particular, has been documented to have a bidirectional relationship with depressive symptoms (Maynard *et al*. 2018). Factors related to perceived needs, including low income and assets, not working, lack of adequate housing, and general insecurity, are associated with poor mental health in conflict-affected populations (Roberts & Browne, 2011). Other risk factors for poor health, such as poverty, malnutrition, education level, and other socioeconomic factors, are not exclusive to conflict settings but are exacerbated due to insecurity, and therefore have a heightened effect in conflict-affected populations (Lorant, 2003; Roberts & Browne, 2011). For instance, before the war, Syria boasted a steadily growing economy, yet northeastern Syria, and specifically Raqqa Governorate, was comprised of semi-arid land primarily used for herding and scattered agriculture and held a concentration of the country's impoverished (World Bank Group, 2017). Food insecurity was also rife pre-conflict, as nearly one in five households in Syria had estimated expenditures on food below the costs of minimum caloric requirements (Nasser *et al*. 2013). These areas, where the current study took place, may have been particularly susceptible to these pre-migration economic risk factors identified in this previous study as salient risk factors for poor mental health. In addition to potentially being more susceptible to poor mental health due to these pre-existing factors in general, armed conflict may also have magnified their associations with depressive symptoms. Estimates suggest that Syria's gross domestic product has been halved since the start of the conflict (Gobat & Kostial, 2016), making the needs to address daily stressors, such as food insecurity and other perceived needs, more pressing to address in and of themselves, as well as their secondary potential to improve mental health.

Food security and economic stressors are also related to household gender dynamics and levels of IPV, which are explanatory factors for the higher prevalence of poor mental health amongst women (Vyas & Watts, 2009). For instance, women reporting unequal household dynamics in Afghanistan were more likely to experience physical and emotional IPV and reported higher levels of food insecurity (Gibbs *et al*. 2018*a*), demonstrating the relationship between economic security, IPV, and mental health for women in emergency settings.

Importantly, disability was also a salient risk for increased depressive symptoms in all models. Globally, this has been consistently linked to poor mental health (Help Age & Handicap International, 2014; Hassan *et al*. 2016). However, the measure used to assess disability has limited specificity in parsing the various forms of disability the women may have been reporting and there may be unintended overlap between disability measures and depressive symptoms (e.g. difficulty caring for oneself may concur with PHQ-9 items). Thus, further research is needed in this area amongst conflict-affected populations as disability status may be an important factor to consider in mental health programming efforts to better meet the needs of women.

Notably, these analyses found that current displacement status was not associated with heightened depressive symptoms, which suggests that both host communities, whom also underwent occupation by ISIS and grapple with ongoing remnants of the civil conflict, and internally displaced people residing in these communities from other parts of Syria, had minimal differences for this mental health outcome. As data are only drawn from communities, we are unable to make inferences about differences in depressive symptoms between internally displace camp and non-camp-based displaced populations.

Limitations of the study include its cross-sectional nature such that causality cannot be inferred and it may be possible that depressive symptoms have a bidirectional relationship with key predictor variables such as food insecurity and IPV. There are several limitations related to self-reporting bias. Experiences of violence and mental health status are often subject to under-reporting due to their sensitive nature. Alternatively, food insecurity and perceived needs may be over-reported as the survey was administered through a non-governmental organization working in the region; it is therefore possible respondents increased reporting of these items if they thought it may have resulted in increased aid. Additionally, the analyses draw from a subsample of married participants participating in a cash transfer program; thus, findings cannot be extrapolated to women with different marital status in the region. In addition, other important predictors such as loss of a loved one or previous history with depression were not captured in the present survey. Finally, the internal consistency reliability of the primary outcome of the analysis, PHQ-9 was acceptable, but may have introduced statistical imprecision into model estimates.

Overall, study findings point to the need to address mental health issues amongst women currently residing in conflict-affected areas in northeastern Syria. Efforts, including cash transfer programming or other longer term economic programming that contribute to reducing daily stressors may alleviate symptoms of depression. Programming such as case management services for survivors which address experiences of IPV may also be helpful in decreasing depressive symptoms in this population. Finally, there is a need to adapt and implement targeted mental health interventions for women with high levels of depressive symptoms, traumatic experiences, and ongoing daily stressors (Miller & Rasmussen, 2010; Bass *et al*. 2013). Such attention to depression, experiences of IPV, as well as daily stressors is urgently needed to promote resilience and eventual recovery in an environment grappling with multiple crises.
